# Automated detection of aggressive and indolent prostate cancer on magnetic resonance imaging

**DOI:** 10.1002/mp.14855

**Published:** 2021-05-03

**Authors:** Arun Seetharaman, Indrani Bhattacharya, Leo C. Chen, Christian A. Kunder, Wei Shao, Simon J. C. Soerensen, Jeffrey B. Wang, Nikola C. Teslovich, Richard E. Fan, Pejman Ghanouni, James D. Brooks, Katherine J. Too, Geoffrey A. Sonn, Mirabela Rusu

**Affiliations:** ^1^ Department of Electrical Engineering Stanford University Stanford CA 94305 USA; ^2^ Department of Radiology Stanford University School of Medicine Stanford CA 94305 USA; ^3^ Department of Urology Stanford University School of Medicine Stanford CA 94305 USA; ^4^ Department of Pathology Stanford University School of Medicine Stanford CA 94305 USA; ^5^ Department of Urology Aarhus University Hospital Aarhus Denmark; ^6^ Stanford University School of Medicine Stanford CA 94305 USA; ^7^ Department of Radiology VA Palo Alto Health Care System Palo Alto CA 94304 USA

**Keywords:** aggressive vs. indolent cancer, deep learning, Gleason grading, prostate MRI

## Abstract

**Purpose:**

While multi‐parametric magnetic resonance imaging (MRI) shows great promise in assisting with prostate cancer diagnosis and localization, subtle differences in appearance between cancer and normal tissue lead to many false positive and false negative interpretations by radiologists. We sought to automatically detect aggressive cancer (Gleason pattern ≥ 4) and indolent cancer (Gleason pattern 3) on a per‐pixel basis on MRI to facilitate the targeting of aggressive cancer during biopsy.

**Methods:**

We created the Stanford Prostate Cancer Network (SPCNet), a convolutional neural network model, trained to distinguish between aggressive cancer, indolent cancer, and normal tissue on MRI. Ground truth cancer labels were obtained by registering MRI with whole‐mount digital histopathology images from patients who underwent radical prostatectomy. Before registration, these histopathology images were automatically annotated to show Gleason patterns on a per‐pixel basis. The model was trained on data from 78 patients who underwent radical prostatectomy and 24 patients without prostate cancer. The model was evaluated on a pixel and lesion level in 322 patients, including six patients with normal MRI and no cancer, 23 patients who underwent radical prostatectomy, and 293 patients who underwent biopsy. Moreover, we assessed the ability of our model to detect clinically significant cancer (lesions with an aggressive component) and compared it to the performance of radiologists.

**Results:**

Our model detected clinically significant lesions with an area under the receiver operator characteristics curve of 0.75 for radical prostatectomy patients and 0.80 for biopsy patients. Moreover, the model detected up to 18% of lesions missed by radiologists, and overall had a sensitivity and specificity that approached that of radiologists in detecting clinically significant cancer.

**Conclusions:**

Our SPCNet model accurately detected aggressive prostate cancer. Its performance approached that of radiologists, and it helped identify lesions otherwise missed by radiologists. Our model has the potential to assist physicians in specifically targeting the aggressive component of prostate cancers during biopsy or focal treatment.

## 1 INTRODUCTION

Prostate cancer is the most frequently diagnosed cancer in American men, with an estimated 191 930 new cases expected in 2020.^1^ Accurate diagnosis and localization of cancer in the prostate is critical for targeted biopsy, monitoring disease for patients on active surveillance, and guiding local treatments.^2^ While multi‐parametric magnetic resonance imaging (MRI) is increasingly used as a noninvasive aide in prostate cancer diagnosis and tumor localization, both false positive and false negative findings remain common, even when using the Prostate Imaging‐Reporting and Data System (PIRADS)^3^, ^4^ reporting scheme.^5^ Ideally, a biopsy would only be performed when cancer is identified on MRI, and only those areas of high suspicion for aggressive cancer would be targeted. This would reduce the morbidity of biopsy and make the results more reliable. However, false positive findings on MRI often lead to unnecessary biopsies in men without cancer, while false negatives lead to extensive biopsy procedures due to fear of missing significant cancers. To realize the full potential of MRI to improve prostate cancer diagnosis while reducing morbidity from biopsy, improvements are needed in the performance and interpretation of MRI by radiologists.^5^

**Fig. 1:**
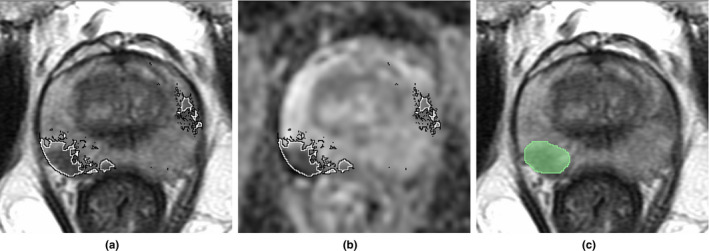
Lesions outlined on MRI often underestimate cancer size while missing some other cancers entirely. One slice of a radical prostatectomy case with (a) T2‐weighted MRI and (b) ADC overlaid with labels obtained from histopathology images (black) compared to (c) T2‐weighted MRI overlaid with radiologist labels (green). Note the hardly visible MRI lesions (patient left) that are missed by the radiologist.

The need for improvement in prostate MRI interpretation has led to interest in using machine learning methods. Both semi‐automated^6^, ^7^, ^8^, ^9^, ^10^ and fully automated^11^, ^12^, ^13^, ^14^, ^15^ computational approaches have been applied to facilitate prostate cancer identification on MRI. These studies typically derive labels from radiologist‐outlined lesions confirmed by biopsy^8^, ^10^, ^15^, ^16^, ^17^ or from cognitive registration of preoperative MRI and histopathology images of the resected tissue for patients undergoing radical prostatectomy.^9^, ^12^, ^13^ All these approaches are dependent on human interpretation of MRI to find all cancer lesions and accurately delineate their extent. Yet, radiologist labels have shortcomings resulting in models (Table I) that fail to capture (a) cancers not visible on MRI (those cancers that cannot be outlined even in the presence of histopathology images from surgery) or (b) cancers that are hardly visible on MRI (those that are missed at initial read, but are observed in retrospect upon review alongside histopathology images from either surgery or biopsy). Such lesions contribute to the 12% of aggressive cancers missed during screening,^18^ and the 34% of aggressive and 81% of indolent cancers missed in men undergoing prostatectomy.^19^ Further complicating these studies is that MRI underestimates lesion size,^20^, ^21^ leading to inaccurate annotations (Fig. 1).

**Table I:** Summary of prior deep learning approaches. Terminology and Abbreviations: Visible MRI Lesions are readily identified by radiologists; Hardly Visible MRI Lesions are found after review of the whole‐mount histopathology images available for surgery patients, MRI Invisible Lesions are lesions that cannot be reliably outlined even in the presence of histopathology images from surgery; Aggressive Cancer (Agg) has Gleason score 3 + 4 and above; Indolent Cancer (Ind) is Gleason Score 3 + 3, Normal is noncancer; CS‐clinically significant cancer; Feats‐Features; Se‐sensitivity; Sp‐specificity; FP‐False Positive; AUC‐ Area Under the Receiver Operating Characteristic Curve.

First author	Label granularity	Evaluation granularity	Task	Architecture	Visibility MRI labels	Without radiologist input	Patient number, split	Performance on Agg and/vs Ind
Cao^12^	Per lesion	Per pixel; per lesion	Gleason score	Modified DeepLabv3	Visible, hardly visible	True	417(fivefold cross val)	AUC (Ind vs Agg): 0.81, FP@80% Se (CS): 0.65, FP@80% Se (All): 2.30
Sanyal^14^	Per lesion	Per pixel	Agg, Ind, normal	UNet	Visible	True	57/20	AUC (Agg): 0.86, AUC (Ind): 0.88
Schelb^17^	Per lesion	Per sextant, per patient	Agg	UNet	Visible, hardly visible	True	250/62	Se (Sextant): 0.59, Sp (Sextant): 0.66, Se (Patient): 0.96, Sp(Patient): 0.50
McGarry^32^	Per pixel	Per lesion	Agg, Ind, normal	Otsu threshold‐ ing	Visible, hardly visible, invisible	True	48 (threefold cross val)/5	AUC (Agg): 0.77, AUC (All Cancer): 0.77
Vente^15^	Per lesion	Per pixel; per lesion	Gleason score	Modified UNet	Visible	True	162 (fivefold cross val)	Dice: 0.37
Yuan^8^	Per lesion	Per patch; per lesion	Agg, Ind	AlexNet transfer learning	Visible	False	111/66/44	AUC (Ind vs Agg): 0.90, Se: 0.87, Sp: 0.88
Zhong^9^	Per lesion	Per lesion	Agg, Ind	ResNet transfer learning	Visible, hardly visible	False	110/30	AUC (Ind vs Agg): 0.76, Se: 0.64, Sp: 0.80
Chaddad^10^	Per lesion	Per lesion; per ROI	Gleason score	Random forest w. CNN feats	Visible	False	99 (fivefold cross val)	AUC (Ind vs Agg): 0.89

An alternative approach to labeling cancer location on MRI is to perform automatic registration of preoperative MRI and digital histopathology images from patients undergoing radical prostatectomy.^22^, ^23^, ^24^, ^25^, ^26^, ^27^ Labels obtained from automatic registration are more accurate than radiologist labels since they do not depend on human interpretation of MRI and allow for the full extent of lesions found on histopathology to be mapped on MRI, including cancers that are invisible or hardly visible. Figure 1 illustrates how cancer labels mapped from histopathology images onto MRI typically extend beyond the radiologist annotation and often include cancers that were not detected by the radiologist. Unlike prior registration studies that included fewer than 50 subjects,^25^, ^26^, ^27^ we have registered histopathology and MR images for over 150 patients at our institution using our RAPSODI platform.^22^ RAPSODI relies on traditional registration methods to identify the optimal affine and deformable transforms between corresponding MR and histopathology images. Moreover, we have recently shown that deep learning methods can accelerate this registration,^23^ while slice‐to‐slice correspondences are not required when using super‐resolution generative adversarial networks to reconstruct 3D histopathology and MRI volumes.^24^ We previously used a subset of the unique dataset generated by RAPSODI^22^ to train a deep learning model to automatically detect prostate cancer on MRI.^11^ Here, we seek to expand upon this work by focusing on distinguishing aggressive from indolent cancers on MRI using labels derived from automated registration of histopathology and MR images. Unlike prior methods that either use radiologist labels or pathology labels mapped from cognitive alignment (radiologists and pathologists jointly reviewing the MR and histopathology images, without computational alignment), our proposed approach is the first to use automatically detected aggressive and indolent cancers on histopathology images^28^ mapped onto MRI to generate labels for aggressive and indolent cancers on MRI.

Previous computational methods to detect aggressive prostate cancer on MRI either (a) relied on hand‐crafted features combined with traditional classification methods^2^, ^6^, ^29^, ^30^ or (b) leveraged deep learning architectures such as the UNet,^14^, ^15^, ^17^ the holistically nested edge detector (HED),^31^ or derived from DeepLab.^12^ The deep learning‐based approaches are summarized in Table I. Some of these approaches are fully automatic, while others require the radiologist to provide regions of interest to be classified by the model. Due to the inaccurate labels used during training or when providing regions of interest (i.e., unable to capture invisible/hardly visible MRI lesions, underestimating cancer extent, and lacking per‐pixel assessments of aggressive and indolent cancers), these methods^12^, ^14^, ^15^, ^17^ are unable to properly detect the true extent of lesions and identify aggressive and indolent cancers when they coexist within the same lesion (Table I). Only the work by McGarry et al.^32^ used pixel‐level labels of cancer obtained in 48 patients from registering histopathology to MR images and were used to create a simple model based on thresholding. Yet, their approach fail to characterize the ability of their model to distinguish different types of cancer coexisting within a lesion. Since our proposed approach uses pixel‐level labels of aggressive and indolent cancer derived from histopathology images mapped onto MRI, we can test whether such a model is able to distinguish aggressive from indolent cancers even when they coexist within the same lesion (known as clinically significant lesions).

Here, we introduce the Stanford Prostate Cancer Network (SPCNet) to distinguish (a) normal tissue, (b) indolent prostate cancer (Gleason pattern 3) and aggressive prostate cancer (Gleason pattern ≥ 4) on multi‐parametric MRI. SPCNet modifies the architecture of the HED Network, a multi‐resolution deep learning architecture, making it a 2.5D network that uses three adjacent slices to capture the volumetric nature of the tumors. Moreover, SPCNet relies on a branched architecture in which separate features are identified for each input MRI sequence, T2‐weighted MRI and apparent diffusion coefficient (ADC). We hypothesize that our deep learning network which learns features specific to each MRI sequence using volumetric context and pixel‐level labels of indolent and aggressive cancers mapped from histopathology images onto MRI is better at predicting prostate cancer compared to alternative approaches, for example, using UNet or DeepLab architectures.

## 2 MATERIALS AND METHODS

### 2.A Dataset

#### 2.A.1 Population characteristics

This retrospective study was approved by the Institutional Review Board (IRB) of Stanford University. As a chart review of previously collected data, we proceeded with a waiver of consent. Our study included patients from two independent cohorts at our institution (Table II). Cohort C1 included 101 patients who had a preoperative MRI examination before undergoing radical prostatectomy, and 30 patients considered to have a normal prostate after having both a negative MRI and a negative biopsy. Cohort C2 included 293 patients who had an MRI examination before undergoing MRI‐Ultrasound fusion targeted prostate biopsy.

**Table II:** Description of our cohorts and data characteristics.

Procedure	Cohort C1		Cohort C2
	Radical prostatectomy	Normal	Biopsy
Number of patients	101	30	293
T2w			
Repetition time (TR, range) (s)	3.9, 6.3	1.7, 7.6	2.0, 7.4
Echo time (TE, range) (ms)	122, 130	81, 149	92, 150
Pixel size (Range) (mm)	0.27, 0.94	0.35, 0.43	0.39, 0.47
Distance between slices (mm)	3.00, 4.20	3.00, 4.20	3.00, 4.20
Matrix size	K, L ∈ [256, 512]	K, L ∈ [512]	K, L ∈ [512]
Number of slices	M ∈ [24, 43]	M ∈ [24, 48]	M ∈ [20, 43]
ADC			
b‐values (*s/mm* ^2^)	[0, 50, 800, 1000, 1200]	[0, 25, 50, 800, 1200, 1400]	[0, 25, 50, 800, 1200, 1400]
Pixel size (range) (mm)	0.78, 1.50	0.78, 1.25	0.78, 1.01
Distance between slices (mm)	3.00, 4.50	3.00, 6.00	3.00, 4.60
Matrix size	K, L ∈ [50, 100, 256]	K, L ∈ [256]	K, L ∈ [256]
Number of slices	M ∈ [15, 40]	M ∈ [24, 48]	M ∈ [14, 42]
Labeled regions (patient number)			
Prostate	Yes (101)	Yes (30)	Yes (293)
Radiologist outlined lesions	Yes (29)	Yes (6)	Yes (293)
Pathologist outlined cancer	Yes (101)	No	No
Per‐pixel Gleason Grade^28^	Yes (101)	No	No

Patients from cohort C1 were split between training/validation (n = 102) and testing (n = 29) sets (Table III). The 29 patients in the test set of cohort C1 (C1‐Test) and all the patients in cohort C2 were used only for evaluating SPCNet. These subjects had their MRI read by board‐certified radiologists (Cohort C1‐Test, 11 radiologists, experience ranging between 1 and 40 yr of post‐residency, median 8 yr) as part of routine clinical care. For each case, one of the 11 radiologists outlined the extent of cancer. A detailed description of the 29 subjects in the test set of cohort C1 is provided in Table S1.

**Table III:** Test sets include a subset of patients in cohort C1 and all subjects in cohort C2. Age and PSA reported as median (range), Gleason scores as count (cohort proportion), and as percentage of the entire cohort size within brackets.

Cohort	C1‐Test	C2
Patient number	29	293
Age (years)	63.8 (49‐76)	65 (38‐82)
PSA (ng/mL)	6.8 (3.3‐28.6)	7.1 (0.9‐63.0)
Gleason score (Percentage)		
Normal	6 (21)	133 (45)
3 + 3	‐	42 (14)
3 + 4	12 (41)	58 (20)
4 + 3	6 (21)	32 (11)
Others	5 (17)	28 (10)
Number of lesions	26	232
Lesion type		
Aggressive	22	155
Indolent	4	77
Lesion location		
Peripheral zone	15	154
Transitional zone	7	76
Peripheral & transitional zones	4	0
Anterior stroma	0	2
Lesion volume (mm^3^)	1,857 ± 2,216	1,842 ± 2,647

#### 2.A.2 Image acquisition and alignment

All multi‐parametric MRIs were acquired using surface coils and GE scanners at 3.0 Tesla. Each MRI acquisition included T2‐weighted (T2w) images, diffusion‐weighted images (DWI) used to compute the ADC maps, and dynamic contrast‐enhanced images. SPCNet only used the T2w and ADC images, viewed as a stack of images of size K×L, with M slices (characteristics summarized in Table II). For radical prostatectomy patients in cohort C1, the excised prostates were serially sectioned using customized 3D‐printed molds with slice thickness matching that of T2w images.^33^ All hematoxylin and eosin‐stained (H&E) histopathology images were scanned to generate digital whole‐mount histopathology images. Each digital histopathology image was aligned with the corresponding MR image using the automated affine and deformable registration method, RAPSODI, described by Rusu et al.,^22^ to enable accurate mapping of cancer labels from histopathology images onto MRI.

#### 2.A.3 Labels

The prostate was segmented on T2w images by expert technologists (mean experience = 9 years) and adjusted as necessary by our expert team (GS — urologic oncologist with 9 yr of experience, MR with >10 yr of experience reviewing prostate MRI, and histopathology images). An expert pathologist (CAK) annotated prostate cancer on all digital histopathology images on a per‐pixel basis. Additionally, we used the deep learning method developed by Ryu et al.^28^ to predict pixel‐level Gleason pattern on our histopathology dataset, which was then registered to MRI to create labels for Gleason patterns 3, 4, and 5 for the radical prostatectomy patients in cohort C1. The annotated histopathology images were then registered to MRI, and the pixel‐level labels of aggressive and indolent cancers from histopathology images were mapped onto MRI.

We also obtained radiologist annotations of suspicious lesions and corresponding PIRADS scores. For patients in cohort C2, these radiologist‐annotated lesions had been used to conduct targeted biopsies. Each lesion was labeled with the pathology from biopsy cores directed at that lesion. When multiple cores from the same lesion showed cancer, the highest Gleason score was assigned to the entire lesion. In the radical prostatectomy cohort, we defined clinically significant lesions based on the amount of aggressive cancer found in the 3D stack of histopathology images that were reconstructed and registered to the MRI. We evaluated our algorithm by defining clinically significant lesions using two criteria: (a) having at least 1% of their pixels labeled as aggressive cancer or (b) having at least 5% of their pixels labeled as aggressive cancer. For cohort C2, lesions with biopsy pathology Gleason Score ≥ 3 + 4 were considered clinically significant cancer.

### 2.B MRI preprocessing

Multiple preprocessing steps were applied to the MRI scans.

1. ADC maps and T2w images were manually registered using affine transformations for the patients in cohort C1. No registration was performed for the studies in cohort C2.
2. MRIs were resampled to the same spacing (0.29 mm × 0.29 mm) and cropped to 224 × 224 pixels centered around the prostate.
3. An intensity standardization method^34^ was applied to align the histogram of the MRI sequences as they can vary across patients and scanners. The process involved (a) independently learning a set of intensity histogram landmarks for each MRI sequence from the entire training dataset, and (b) transforming the image histograms to align with the mean histogram of the MRI sequence learned in step (a). The intensity standardization method was applied to the prostate pixels for both T2w and ADC images independently. Since model training involved splitting the data into training and test sets, we learned the histogram average in the training set of cohort C1 and used it to align the cases in the test sets from both cohorts C1 and C2.
4. Input samples were normalized such that pixels within the prostate had a mean of 0 and a standard deviation of 1.

### 2.C SPCNet

**Fig. 2:**
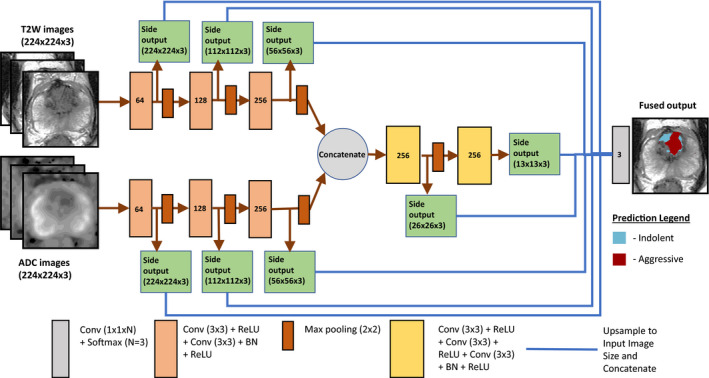
The SPCNet architecture.

We propose a new convolutional neural network model named Stanford Prostate Cancer Network (SPCNet). This network is based on the holistically nested edge detector (HED) architecture^35^ used in previous prostate cancer detection work.^11^, ^13^ Similar to the HED, SPCNet has multiple outputs at various image scales, which are then upsampled and fused to form the final output (Fig. 2). The network is designed to distinguish between three classes: (a) normal tissue, (b) indolent cancer and (c) aggressive cancer, and takes as input bi‐parametric MRI, that is, T2w images and ADC maps, to produce pixel‐level probabilities of the three classes.

Unlike the HED, SPCNet uses three adjacent slices of multi‐parametric MRI to predict cancer on the middle slice and has separate convolutional layers for each MRI sequence before concatenating their outputs and predicting cancer (Fig. 2). By including three adjacent slices, SPCNet incorporates volumetric information from the adjacent slices when predicting the presence of cancer. The use of separate parameters and outputs for each imaging component at larger scales before concatenating them for the smaller scale features seeks to have the model learn features unique to each component at larger scales.

### 2.D Training

We trained SPCNet using fivefold cross‐validation with the patients in cohort C1 (n = 102), and tested our model on a held‐out set from cohort C1 (C1‐Test, n = 29) and the entire set of patients in cohort C2 (n = 293, Table III). For radical prostatectomy patients, only the slices with cancer were coupled with their adjacent slices and used during training. SPCNet was trained for 25 epochs with a batch size of 32 using the Adam optimizer with a learning rate of $10^{‐3}$. Training data were augmented with random rotation ranging between −15 and 15 degrees and left to right flipping.

Labels from both the expert pathologist and grade information from Ryu et al.^28^ were used to train SPCNet. Pixels labeled either Gleason pattern 4 or 5^28^ were considered aggressive and pixels labeled Gleason pattern 3^28^ were considered indolent regardless of the pathologist label. Pixels labeled by the expert pathologist without any grade information were considered either aggressive or indolent with an equal likelihood of 0.5. These disagreements between the expert pathologist and the grade information labels were rare and typically consisted of a small number of pixels for each patient. Finally, pixels with no cancer label from either source were labeled as normal tissue.

The loss function used to train SPCNet was a weighted version of the categorical cross‐entropy that weighs pixels from the three classes by the inverse proportion of pixels of each class computed across the entire training set. This loss function is mathematically represented by Equation 1 and Equation 2 where a pixel's ground truth label is given by $\left[y_{1},y_{2},y_{3}\right]$ and its prediction is given by $\left[{\hat{y}}_{1},{\hat{y}}_{2},{\hat{y}}_{3}\right]$. There are M pixels in the training set and N pixels in a batch with $y^{m}$ as the label of the *m*th pixel in the training set and $y^{n}$ as the label of the *n*th pixel in a batch.

(1) $$‐\frac{1}{N}\sum_{n=1}^{N}\sum_{i=1}^{3}w_{i}y_{i}^{\left(n\right)}\ln\;{\hat{y}}_{i}^{\left(n\right)}$$

where

(2) $$w_{i}=\frac{M}{\sum_{m=1}^{M}y_{i}^{\left(m\right)}}$$

### 2.E Prior networks

In addition to training SPCNet, we also trained alternative models using the UNet^14^, ^36^ and DeepLabv3+^37^ architectures as baselines for prior approaches. Since the source code was not available for either of the architectures for their respective prostate cancer studies, we implemented the versions made available by Ronneberger et al.^36^ and Chen et al.,^37^ respectively. The only modifications included increasing the number of output layers to three to accommodate the multiclass problem. Similar to SPCNet, we trained these models using the previously described augmentation and loss function for 25 epochs using a batch size of 32 with the Adam optimizer. Both UNet and DeepLabv3+ used a single slice input for each MRI sequence and a learning rate of $10^{‐6}$.

### 2.F Evaluation

We evaluated our model using several approaches. First, we performed our evaluation on a per‐pixel basis, as is commonly done for segmentation problems. Then, we performed per‐lesion and per‐patient evaluations, which are more relevant to clinical settings. However, there is no universally agreed‐upon method for evaluating a model on a per‐lesion or per‐patient level. Moreover, the cancer labels projected from histopathology images had to be processed in order to create lesion outlines from small and separate regions.

We quantitatively evaluated our models in two cohorts: (a) the test set from cohort C1 (C1‐Test), and (b) all patients in cohort C2. To evaluate SPCNet in the cohorts C1‐Test and C2, we averaged the outputs of the five models resulting from the cross‐validation to create one probability map and then computed metrics on the average prediction results.

#### 2.F.1 Per‐pixel evaluation

To be consistent with data in the training set, we only evaluated slices that had been annotated as having cancer in radical prostatectomy and biopsy patients. We concatenated the predicted probability of cancer for all pixels within the prostate for all cases and computed the area under the receiver operating characteristic curve (AUC ROC). We then thresholded the predicted probability to compute sensitivity and specificity, which are common classification metrics defined as

(3) $$Sensitivity=\frac{True\;Positives}{True\;Positives+False\;Negatives}$$

and

(4) $$Specificity=\frac{True\;Negatives}{True\;Negatives+False\;Positives}$$

These thresholds were chosen through empirical testing and were found to be reliable for detecting lesions on MRI.

The three classes for SPCNet were evaluated individually on a one vs all manner. Pixels that were annotated by the pathologist but lacked grade information were excluded in the evaluation of aggressive and indolent pixels.

#### 2.F.2 Lesion outlines

For the radical prostatectomy patients in cohort C1, we processed the cancer labels projected from histopathology images by applying a three‐dimensional morphological closing operation. This allowed us to connect separate label regions that are small and close together into distinct, connected lesions throughout the 3D volume. Finally, we computed the effective volume of these lesions and discarded lesions with volumes below 250 mm^3^ for the lesion‐ and patient‐level evaluations (see below). We selected this threshold because such small volume lesions ($\approx 6\times 6\times 6\;{\text{mm}}^{3}$, seen at most on two consecutive MRI slices) have previously been regarded as clinically insignificant.^38^ We also verified that this threshold was less than the threshold used by the PIRADS reporting scheme to define clinically significant cancer (≥500 mm^3^).^3^, ^4^ Our thresholding makes the lesion volume threshold lower than that of most other studies which used radiologist lesions as ground truth. Supplementary S1 demonstrates that our threshold only removed two lesions with relatively small volumes. This process allowed us to generate lesion borders from our pathology annotations to be used to perform lesion‐level analysis on MRI for our models. This thresholding was not applied to the training data, and does not affect the trained model.

#### 2.F.3 Per‐lesion evaluation

**Fig. 3:**
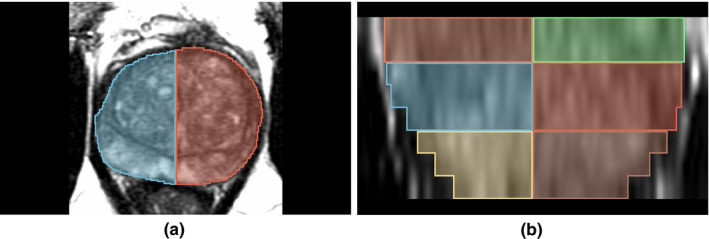
Axial (a) and Coronal (b) views of the sextants (one color per sextant).

We used connected component analysis on the morphologically processed pathology labels to extract the individual lesions to serve as our ground truth. For true positives and false negatives, a lesion was considered detected if the 90th percentile of probabilities from the model within the lesion outline exceeded a threshold. For true negatives and false positives, we split the entire prostate into sextants by dividing the prostate into left and right regions and then splitting these halves into three regions along the longitudinal axis (Fig. 3). This division of the prostate follows the standard approach of systematic prostate biopsy. We considered the sextants in which ≥95% of pixels were benign as the ground truth for negatives. As with the lesion outline, the 90th percentile of model probabilities in the sextant was used to determine whether the model classified it as a true negative or false positive. With the paradigm for true positives and true negatives established, we computed ROC AUC, sensitivity, and specificity using the same thresholds from the per‐pixel analysis. This per‐lesion analysis was used to evaluate the detection of all cancer lesions as well as clinically significant lesions. For all cancer lesions, we used the predicted probability of any cancer, and for clinically significant lesions we used the predicted probability for aggressive cancer.

#### 2.F.4 Per‐patient evaluation

We performed a patient‐level evaluation in the cohort C1‐Test, using cases with clinically significant lesions to determine the true positives and false negatives. Moreover, we used normal cases to determine true negatives and false positives. For patients with clinically significant lesions, a patient was classified a true positive if the model was able to detect at least one of the clinically significant lesions or a false negative if the model could not detect any of the clinically significant lesions. To determine if a lesion is detected, the same procedure from per‐lesion evaluation is used. For normal patients, SPCNet's prediction was thresholded and morphologically processed to define predicted lesions. The thresholds used were derived from per‐pixel analysis and the morphological processing following the same procedure used to define lesion outlines from the ground truth labels. If there was a predicted lesion on a normal case then it was classified as a false positive, otherwise it was classified as a true negative. Since this procedure involved hard thresholding and not probability values, only sensitivity and specificity were computed.

#### 2.F.5 Radiologist comparison

Finally, we compared SPCNet with the radiologists at a lesion and a patient level in the cohort C1‐Test. However, such analysis was not performed in cohort C2 as our labels are derived from radiologists. For these comparisons, we evaluated the radiologist outlines and model predictions the same way. Because PIRADS v2.1 is designed to specifically detect clinically significant cancer, radiologist outlines were treated as a prediction where every pixel within their outline was predicted to be aggressive cancer with a probability of 1.00. We only computed sensitivity and specificity since the ROC AUC would not be comparable. Additionally, we evaluated a combination of the model predictions and radiologist outlines by adding them to gain insight into the potential performance of a radiologist assisted by our model.

## 3 RESULTS

**Fig. 4:**
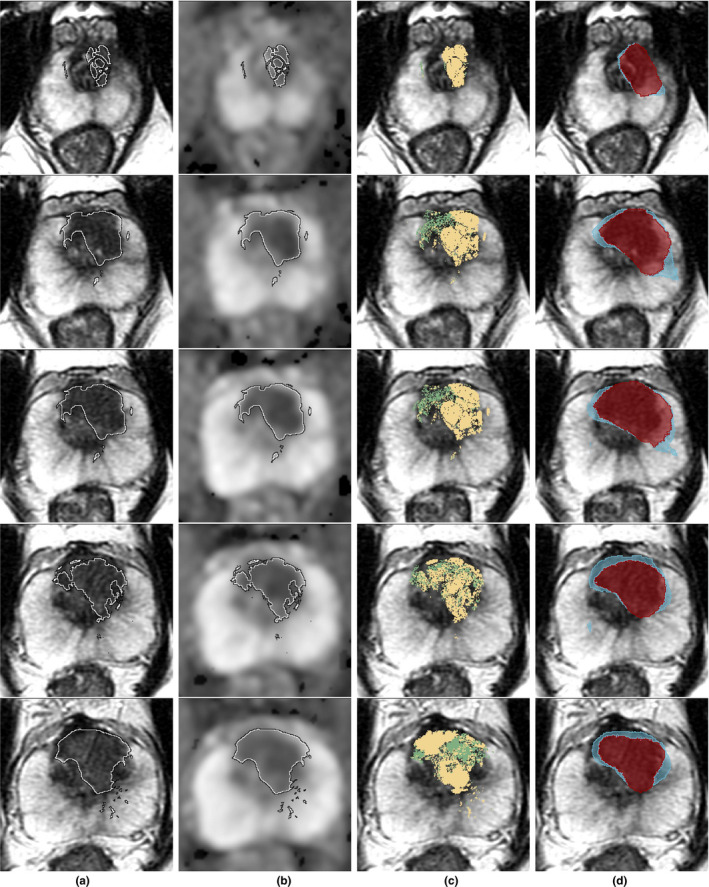
SPCNet predictions on a sample patient in cohort C1‐Test shown from apex (top row) to base (bottom row). The per‐pixel AUCs were 0.92 for normal tissue, 0.90 for indolent cancer, and 0.93 for aggressive cancer. (Column a) T2w input; (Column b) ADC; (Column c) T2w image overlaid with grade annotations Indolent (Gleason pattern 3, Green) and Aggressive (Gleason patterns 4 or 5, Yellow); (Column d) T2w image overlaid with thresholded prediction from the SPCNet multiclass model cancer predictions (indolent — blue; aggressive — red). Cancer labels by the expert pathologist mapped from histopathology images onto MRI are outlined in black and white (Columns a–b).

**Fig. 5:**
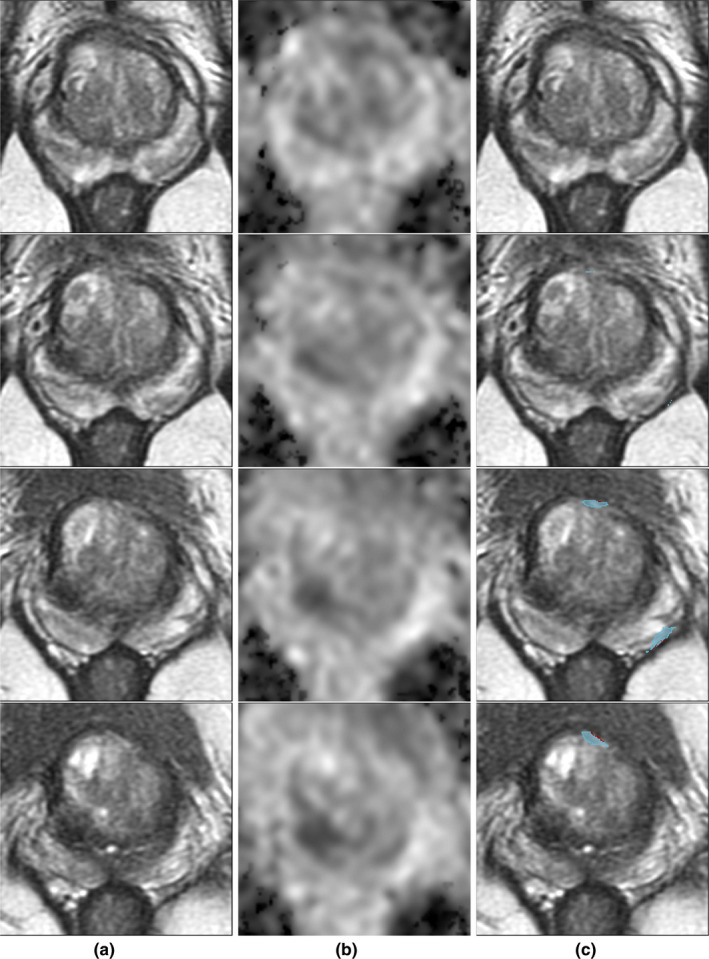
Indolent and aggressive cancer predictions on a sample patient without cancer in cohort C1‐test shown from apex (top row) to base (bottom row). The evaluation showed a per‐pixel accuracy of 0.96 for normal tissue. (Column a) T2w input; (Column b) ADC; (Column c) T2w image overlaid with thresholded prediction from the SPCNet multiclass model cancer predictions (indolent — blue; aggressive — red).

SPCNet accurately detected the extent of indolent and aggressive cancer within the lesion (Fig. 4) and accurately detected normal tissue within patients without cancer (Fig. 5). The quantitative evaluation in the two cohorts, C1‐Test and C2, showed that SCPNet achieves an AUC of 0.80–0.81 to detect normal tissue, 0.64–0.75 to detect indolent cancer, and 0.86–0.89 to detect aggressive cancers at pixel‐level (Table IV) and an AUC of 0.75–0.80 to detect clinically significant lesions (Table V).

**Table IV:** Per‐pixel evaluation.

Model	Class	C1‐test			C2		
		AUC	Se	Sp	AUC	Se	Sp
SPCNet	Normal	**0.80**	**0.93**	0.47	**0.81**	**0.92**	0.56
	Indolent	**0.75**	0.52	**0.83**	**0.64**	0.41	**0.82**
	Aggressive	**0.89**	**0.59**	**0.96**	**0.86**	0.60	**0.93**
UNet	Normal	0.66	0.71	0.54	0.71	0.64	**0.69**
	Indolent	0.64	0.54	0.69	0.63	**0.58**	0.62
	Aggressive	0.68	0.57	0.72	0.74	**0.72**	0.65
DeepLabv3+	Normal	0.60	0.55	**0.61**	0.58	0.54	0.57
	Indolent	0.60	**0.59**	0.57	0.54	0.43	0.62
	Aggressive	0.53	0.50	0.55	0.57	0.62	0.48

Note: Bold indicates the best performance for each metric.

Abbreviations: AUC–ROC — area under the receiver operator characteristic curve; Se — sensitivity, Sp — specificity.

**Table V:** Per‐lesion evaluation.

Model	Class	C1‐test			C2		
		AUC	Se	Sp	AUC	Se	Sp
SPCNet	All	**0.76**	0.62	**0.83**	**0.75**	0.67	**0.75**
	CS	**0.75**	0.50	**0.81**	**0.80**	0.70	**0.77**
UNet	All	0.52	0.81	0.04	0.66	0.95	0.06
	CS	0.56	0.82	0.09	0.66	0.94	0.07
DeepLabv3+	All	0.55	**0.92**	0.01	0.50	**0.96**	0.00
	CS	0.45	**1.00**	0.01	0.65	**0.99**	0.01

Note: Bold indicates the best performance for each metric.

Abbreviations: AUC–ROC — area under the receiver operator curve; Se —— sensitivity, Sp — specificity.

In addition to SPCNet, we also trained alternative networks based on UNet and DeepLabv3+ to distinguish normal tissue, indolent cancer, and aggressive cancer on prostate MRI (Tables IV, V). SPCNet overall achieves higher AUCs than UNet or DeepLabv3+. While the sensitivity of UNet or DeepLabv3+ can be higher than that obtained by SPCNet, the specificity was so low that those models would not be helpful in a clinical setting (<0.01 in Table V). UNet and DeepLabv3+ appear less effective at accurately differentiating cancer types on a per‐pixel basis. These results illustrate that SPCNet is better at detecting and distinguishing aggressive and indolent cancer on MRI (based on both the per‐pixel and per‐lesion evaluations).

SPCNet achieved a similarly large per‐pixel AUC for normal tissue and aggressive cancer across cases in both C1‐Test and C2. However, this trend did not extend to indolent cancer which had a noticeably lower ROC AUC when comparing C1‐Test and C2. This is likely due to the inaccurate labels used for cohort C2 where every pixel in a lesion was considered indolent if the biopsy core associated with it was indolent. Another interesting per‐pixel result is that the sensitivity and specificity for normal tissue were very different since normal tissue has the opposite class imbalance compared to cancer. On a per‐lesion basis, SPCNet performed comparably across both cohorts for detecting all cancer lesions but performed worse on C1‐Test when detecting clinically significant lesions.

Finally, we compared SPCNet with radiologists by assessing their sensitivity and specificity in detecting clinically significant lesions and at a patient level for the subjects in cohort C1‐Test. In addition, we created a combined model that summed the SPCNet and radiologist predictions to evaluate the potential impact of SPCNet on the radiologist interpretation. Table VI shows that SPCNet approaches the performance of radiologists, while finding 13–18% of clinically significant lesions otherwise missed by radiologists. Moreover, the combined model had the best sensitivity, suggesting the utility of using deep learning‐based models to improve the interpretation of prostate MRI. Visual inspection indicated that the lesions detected by SPCNet but not by radiologists had a more subtle appearance on T2w images and were more distinctive on ADC images. The ability of SPCNet to detect lesions with more subtle appearance on T2w images does come at a cost in the form of a lower specificity compared to that of the radiologist.

**Table VI:** Comparing SPCNet and Radiologists for detecting clinically significant lesions on MRI in cohort C1‐Test. Abbreviations: Se — sensitivity, Sp — specificity.

Model	Aggressive threshold	Per lesion		Per patient	
		Se	Sp	Se	Sp
SPCNet	1%	0.50 (11/22)	0.81	0.56 (10/18)	0.83 (5/6)
	5%	0.53 (8/15)	0.81	0.57 (8/14)	0.83 (5/6)
Radiologist	1%	0.59 (13/22)	0.98	0.72 (13/18)	1.00 (6/6)
	5%	0.67 (10/15)	0.94	0.71 (10/14)	1.00 (6/6)
Combined	1%	0.81 (17/22)	0.79	0.89 (16/18)	0.83 (5/6)
	5%	0.80 (12/15)	0.78	0.86 (12/14)	0.83 (5/6)

## 4 DISCUSSION

We developed a new convolutional neural network (SPCNet) and trained it using aggressive and indolent cancer labels mapped from histopathology images onto MRI for patients who underwent radical prostatectomy. Our study had four key findings. First, we found that SPCNet successfully localized and distinguished indolent and aggressive cancer. Second, we found that SPCNet performed comparably across cohorts ranging from patients without cancer to those with early or advanced cancer (Cohort C1: 23 patients who underwent radical prostatectomy, six patients with normal prostates; Cohort C2: 293 patients who underwent MRI‐targeted biopsy). Third, we found that SPCNet approaches the performance of radiologists. Fourth, we found that SPCNet outperforms previously used networks, that is, UNet and DeepLabv3+.

SPCNet achieved a considerably higher sensitivity in detecting clinically significant lesions on cohort C2 compared to cohort C1‐Test. This higher performance may be the result of the difference in definition of clinically significant lesions between the two cohorts. The clinically significant lesions in cohort C2 were lesions initially detected by radiologists on MRI, that is, MRI visible lesions, while some of the lesions in the cohort C1‐Test are invisible or hardly visible on MRI. Hence, a fair evaluation involves comparing the performance of SPCNet and radiologists on the patients in cohort C1‐Test.

SPCNet approached, but did not surpass, the sensitivity or specificity of the radiologists. SPCNet detected fewer lesions than radiologists, while finding up to 18% of clinically significant lesions otherwise missed by the radiologists. These lesions tended to have a subtle T2w appearance, were often localized in the peripheral zone, and would have been missed without either the ground truth from the resected prostate or the prediction of SPCNet. However, this phenomenon led to a lower specificity compared to that of the radiologist due to the higher sensitivity to lesions with subtle T2 appearance. These results along with the performance of the combined model (where radiologist and SPCNet predictions are added) suggest the value of using deep learning models to improve the interpretation of MRI. However, to fully appreciate the impact of SPCNet in clinical settings, further investigation must be conducted. SPCNet was effective in detecting cancer lesions and clinically significant lesions on patients who underwent either radical prostatectomy or biopsy, suggesting the generalizability of our approach within data from our institution.

Our approach has several novel contributions. First, we labeled the MR images using automatically registered histopathology images combined with automated Gleason grading. This enabled us to label each pixel from an MRI with its corresponding histopathology information. Second, we trained a deep learning network to distinguish aggressive and indolent cancer on a per‐pixel basis on MRI, while previous studies have evaluated their approach on a per‐lesion basis.^12^, ^15^, ^22^ This is particularly important from a clinical perspective because preferential detection of aggressive cancer is the widely accepted goal of prostate cancer diagnosis. Third, we considered data from both patients who underwent radical prostatectomy and patients who underwent biopsy. This suggests that SPCNet generalizes outside of the patient cohort used for training to other patients from our institution who were imaged on similar MRI scanners. Fourth, we evaluated our SPCNet model for detecting aggressive and indolent prostate cancer on MRI at pixel, lesion, and patient levels, respectively. While the per‐pixel analysis is straightforward to calculate, the per‐lesion and per‐patient evaluations have higher clinically relevance. Our contributions pave the way for more accurate models that can distinguish different types of cancer on a per‐pixel basis.

A consequence of the novelty of our work is that it makes comparisons to previously published work difficult. As shown in Table I, all previous deep learning methods used labels that lack the granularity of our labels while failing to capture either MRI invisible or hardly visible lesions, or both. Prior automated methods using deep learning models^12^, ^14^, ^15^, ^17^ were trained and evaluated with data similar to cohort C2 and cannot be directly compared to SPCNet. Training with labels from radiologists creates a model that can only detect lesions already detected by radiologists while training with labels from histopathology images allow for models to detect lesions missed by radiologists which is a more challenging task. The fact that SPCNet is better at detecting clinically significant lesions on cohort C2 compared to cohort C1‐Test (despite being trained with cases from cohort C1) suggests that indeed, detecting clinically significant lesions in cohort C2 is an easier task. SPCNet is the first model to be trained and evaluated for the more difficult task of detecting all lesions irrespective of whether they are MRI visible or invisible.

Lack of public access to the networks or code from prior deep learning models^12^, ^14^, ^15^, ^17^ prevented us from directly evaluating prior deep learning models in our test sets. However, we did our best to represent these methods using the DeepLabv3+^12^ and UNet^14^, ^15^, ^17^ architectures. SPCNet outperformed both UNet‐ and DeepLabv3+‐based architectures.

Our approach has a few limitations. First, our training cohort was relatively small (n = 102). This is a consequence of our dataset being the first of its kind with unique pixel‐level labels of aggressive and indolent cancer. However, the consistent performance of SPCNet across 322 patients demonstrates that the size of the training set did not significantly impact how well SPCNet can generalize. Future work will focus on increasing the size of our training cohort even further. Second, despite our rigorous process of labeling the data, the labels for cohort C1 used during training and evaluation are imperfect. For example, the registration of histopathology and MR images has been shown to have a misalignment error of 2 mm on the prostate border and 3 mm inside the prostate.^22^ Due to these known registration errors, we discard very small lesions, which are more affected by these errors but also less clinically relevant. Despite these registration errors, labels obtained from registering histopathology are preferable to labels used by previous work due to their independence from human interpretation of MRI. Accurate per‐pixel predictions of aggressive and indolent cancers on MRI would be tremendously valuable for guiding biopsy and treatment. However, this is unavailable using currently available MRI interpretation techniques. We assessed our ability to provide this information using per‐pixel experiments which are adversely affected by our label registration errors. We also assessed the ability to detect clinically significant lesions. This clinically important task is less affected by registration errors. Third, our Gleason pattern labels came from a deep learning model instead of expert genitourinary pathologists. While it is impractical to have pathologists identify and grade all cancer pixels on large number of cases, the deep learning model has shown excellent results in cancer grading^28^ and its results were combined with the cancer outlines provided by the genitourinary pathologist. Fourth, unlike radiologists in clinical practice, we do not incorporate clinical features such as PSA, gland size, and prior biopsy status that have been shown to improve the predictive accuracy of MRI. We expect that incorporation of these data in future work may further improve model accuracy. Fifth, our study used retrospective data and has not attempted to evaluate the effect of using SPCNet in clinical settings to assist radiologists in their MRI interpretation. Our preliminary results suggest that SPCNet is able to detect clinically significant lesions that radiologists missed, prompting us to consider future prospective studies to fully understand the potential of SPCNet when assisting radiologists. Finally, all studies were obtained from a single institution and one MRI manufacturer. It is likely that testing on scans obtained from outside institutions and on scanners from other vendors will demonstrate suboptimal performance. Future work will incorporate training and testing data from other sites.

Despite these limitations, our model shows great promise. Selective identification of aggressive prostate cancer on MRI would have tremendous clinical value given that the primary goal of prostate cancer early detection is to identify and treat aggressive cancer while reducing overdetection and overtreatment of indolent cancer. Prior deep learning publications have not attempted to specifically find aggressive prostate cancer on a per‐pixel basis, in large part due to the lack of gold standard training data identifying the location and extent of aggressive cancer. The fact that our model can both detect clinically significant cancer as well as localize the aggressive component suggests that it may help urologists to target biopsies at the highest yield locations and spare some men with indolent cancer from invasive biopsy. Furthermore, its similar performance to subspecialty radiologists suggests that it may provide clinical value in future for identifying aggressive cancer, even those missed by radiologists, while improving inter‐reader variability. With further improvements, including increasing the quantity and diversity of training data and prospective validation in a clinical setting, this model could have a major impact in patient care.

## 5 CONCLUSIONS

Our study showed that the Stanford Prostate Cancer Network (SPCNet) was able to accurately detect aggressive and indolent cancer on prostate MRI. SPCNet generalized well in patients who either underwent radical prostatectomy or biopsy and approached the sensitivity and specificity of radiologists when detecting clinically significant cancers, including up to 18% of lesions otherwise missed by radiologists. With further improvements in the future, this model could be implemented to help assist radiologists to interpret prostate MRI.

## CONFLICT OF INTEREST

Mirabela Rusu has research grants from GE Healthcare and Philips Healthcare.

## Supporting information

**Table S1**. Details of test set cases from cohort C1. Gleason Patterns 4 and 5 (%) indicate percentage of cancer lesion volume that has Gleason pattern 4 or above. Involved prostate (%) indicates percentage of prostate volume that has cancer. Lesion volumes indicated by ^∗^ (Serial Numbers 19, 20, and 23) were not considered in our lesion‐ and patient‐level evaluations as they are <250 mm^3^.

## Data Availability

The data that support the findings of this study are available from the corresponding author upon reasonable request.
