# Supplementary material for: Automated detection of aggressive and indolent prostate cancer on magnetic resonance imaging
Source: Med Phys. 2021 May 3;48(6):2960–72. doi: 10.1002/mp.14855 (PMC8360053; doi:10.1002/mp.14855)
Supplement: Supplementary file 1 — Table S1. Details of test set cases from cohort C1. Gleason Patterns 4 and 5 (%) indicate percentage of cancer lesion volume that has Gleason pattern 4 or above. Involved prostate (%) indicates percentage of prostate volume that has cancer. Lesion volumes indicated by ∗ (Serial Numbers 19, 20, and 23) were not considered in our lesion‐ and patient‐level evaluations as they are <250 mm3. [file MP-48-2960-s001.docx]

Supplementary Material: Automated Detection of Aggressive and Indolent Prostate Cancer on Magnetic Resonance Imaging

Seetharaman et. al.

Table S1: Details of test set cases from cohort C1. Gleason Patterns 4 & 5 (%) indicates percentage of cancer lesion volume that has Gleason pattern 4 or above. Involved prostate (%) indicates percentage of prostate volume that has cancer. Lesion volumes indicated by *^∗^* (Serial Numbers 19, 20, and 23) were not considered in our lesion and patient level evaluations as they are less than 250 *mm*^3^.

| Serial No. | Radiologist Lesions  (No.) | PIRADS  score | Pathologist Lesions  (No.) | Pathology Lesion  Volumes (mm^3^) | Gleason Score | Gleason Patterns  4 & 5 (%) | Involved Prostate  (Percentage) |
| --- | --- | --- | --- | --- | --- | --- | --- |
| 1 | 0 | - | 0 | - | - | - | - |
| 2 | 0 | - | 0 | - | - | - | - |
| 3 | 0 | - | 0 | - | - | - | - |
| 4 | 0 | - | 0 | - | - | - | - |
| 5 | 0 | - | 0 | - | - | - | - |
| 6 | 0 | - | 0 | - | - | - | - |
| 7 | 1 | 5 | 1 | 589 | 4+3 | 78 | 10 |
| 8 | 1 | 4 | 2 | 959, 1666 | 4+3 | 60 | 10 |
| 9 | 1 | 5 | 1 | 4932 | 4+5 | 90 | 20 |
| 10 | 1 | 5 | 1 | 7937 | 4+5 | 80 | 20 |
| 11 | 1 | 4 | 1 | 480 | 3+4 | 1 | 5 |
| 12 | 1 | 4 | 2 | 2204, 283 | 4+3+5 | 60 | 15 |
| 13 | 1 | 4 | 2 | 341, 642 | 3+4 | 1 | 5 |
| 14 | 0 | - | 1 | 629 | 4+5 | 80 | 5 |
| 15 | 1 | 5 | 1 | 3523 | 4+3 | 60 | 10 |
| 16 | 1 | 4 | 1 | 337 | 3+4 | 5 | 5 |
| 17 | 1 | 5 | 1 | 1860 | 3+4 | 5 | 20 |
| 18 | 2 | 5,4 | 1 | 877 | 3+4 | 10 | 10 |
| 19 | 1 | 4 | 1 | 190*^∗^* | 3+4 | 10 | 3 |
| 20 | 1 | 5 | 1 | 463 | 4+3 | 60 | 5 |
| 21 | 1 | 4 | 1 | 1017 | 4+3 | 60 | 10 |
| 22 | 0 | - | 1 | 68*^∗^* | 3+4 | 40 | 0.5 |
| 23 | 1 | 5 | 1 | 4786 | 4+5 | 80 | 15 |
| 24 | 2 | 3,3 | 1 | 2354 | 3+4 | 10 | 5 |
| 25 | 1 | 5 | 1 | 1651 | 4+5 | 90 | 5 |
| 26 | 0 | - | 1 | 8816 | 3+4 | 30 | 5 |
| 27 | 0 | - | 1 | 1323 | 3+4 | 5 | 3 |
| 28 | 2 | 5,3 | 1 | 2677 | 3+4 | 10 | 15 |
| 29 | 1 | 4 | 3 | 662, 472, 266 | 3+4 | 15 | 10 |

1
